# Polyphenols in the Central Nervous System: Cellular Effects and Liposomal Delivery Approaches

**DOI:** 10.3390/ijms26136477

**Published:** 2025-07-04

**Authors:** Mateusz Kaluza, Dominika Ksiazek-Winiarek, Piotr Szpakowski, Joanna Czpakowska, Julia Fijalkowska, Andrzej Glabinski

**Affiliations:** 1 Department of Neurology and Stroke, Medical University of Lodz, Zeromskiego 113 Street, 90-549 Lodz, Poland; mateusz.kaluza@umed.lodz.pl (M.K.); dominika.ksiazek@umed.lodz.pl (D.K.-W.); joanna.czpakowska@umed.lodz.pl (J.C.); andrzej.glabinski@umed.lodz.pl (A.G.); 2 Medical University of Lodz, T. Kosciuszki 4 Avenue, 90-419 Lodz, Poland; julia.fijalkowska@student.umed.lodz.pl

**Keywords:** polyphenols, CNS, neurodegenerative diseases, neuroprotection, oxidative stress, nanodelivery, lipid-based nanocarriers

## Abstract

Neurodegenerative and neuroinflammatory diseases of the central nervous system are closely linked to aging and sustained oxidative and inflammatory stress. Polyphenols, plant-derived secondary metabolites, exhibit broad biological activities, including antioxidant and anti-inflammatory effects, the modulation of pathways such as PI3K/Akt, MAPK, Nrf2, and CREB, and the regulation of neurogenesis and microglial activation. This review focuses on the cell-specific actions of selected polyphenols in neurons, astrocytes, microglia, and oligodendrocytes within the context of Alzheimer’s disease, Parkinson’s disease, and multiple sclerosis. A major limitation to the therapeutic use of polyphenols is their poor bioavailability, due to instability, low solubility, and limited blood–brain barrier penetration. Liposomal nanocarriers are explored as promising delivery systems to overcome these barriers. Both conventional and functionalized liposomes (e.g., PEGylated, receptor-targeted) are discussed, alongside in vitro and in vivo studies demonstrating enhanced efficacy compared to free compounds. Intranasal delivery is also presented as a viable alternative to oral administration. Overall, polyphenols offer great potential as neuroprotective agents, and liposome-based delivery platforms have the potential to significantly enhance their clinical potential, provided that key formulation and targeting issues are addressed.

## 1. Introduction

Neurological disorders are recognized as a leading cause of physical and cognitive disability, affecting up to 15% of the global population [1]. Advances in medical care and increased treatment efficacy have contributed to a rise in life expectancy. In parallel, many countries are experiencing a steady growth in the elderly population [2,3]. Neurodegenerative diseases, such as Alzheimer’s disease (AD) and Parkinson’s disease (PD), are strongly associated with aging. Patients affected by these conditions often suffer from debilitating symptoms, including cognitive impairment, memory loss, depression, and motor dysfunction. These diseases are multifactorial in origin and remain incompletely understood. Hallmarks of neurodegenerative processes include neuronal dysfunction, the accumulation of cellular damage, and eventual neuronal loss. Two key pathophysiological processes contributing to disease progression are oxidative stress, driven by the overproduction of reactive oxygen species (ROS), and chronic neuroinflammation [4].

**Figure 1:**
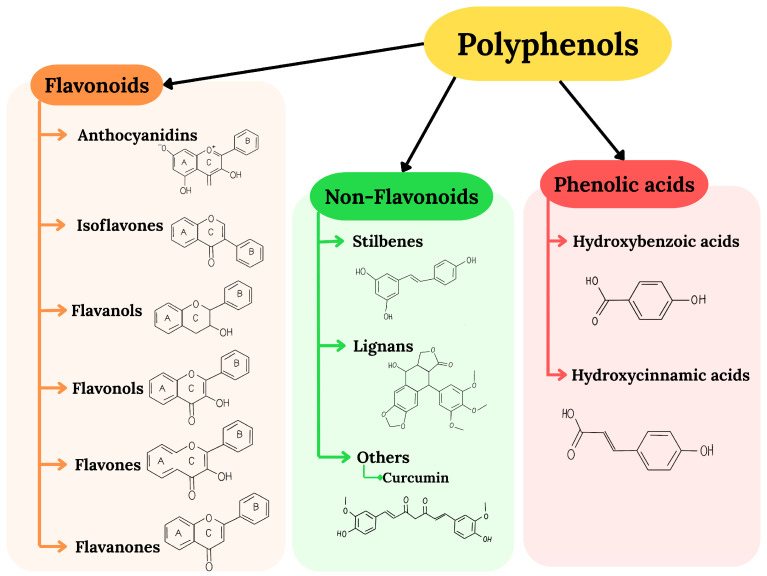
Classification of polyphenols and chemical structures (source of structures PubChem).

In recent years, there has been growing interest in the therapeutic potential of natural compounds, particularly plant-derived bioactives. Among these, polyphenols have emerged as promising candidates due to their wide spectrum of biological activity. Polyphenols constitute a broad and diverse group of secondary metabolites of plants, ubiquitous in commonly eaten foods. For example, polyphenols can be found in berries, grapes, onions, coffee, and wine [5]. It is assumed that the daily intake of phenolic compounds hovers around 1g, which places polyphenols as the first source of antioxidants in the human diet [6]. The categorization of polyphenols is complex and depends on chemical structure, the source of their origin, and biological function. There are more than 8000 compounds assigned to the polyphenols group. The simplest division encompasses flavonoids, non-flavonoids, and phenolic acid [7]. A more detailed classification of these compounds is shown in Figure 1. Notably, polyphenols’ localization in plants is diversified. Water-soluble polyphenols are stored in the vacuoles of plant cells, while water-insoluble polyphenols are stored in the cell walls. Many factors influence the distribution of polyphenols in plants, including processing, storage, part of the plant, environmental factors, and harvest time. For example, polyphenols accumulate more often in the outer layers of plants than in the inner ones [8,9]. Polyphenols are best known for their antioxidant properties. However, accumulating evidence demonstrates that their biological activity is multi-directional. Polyphenols modulate key cellular signaling pathways, such as protein kinase B (Akt), nuclear factor erythroid 2-related factor 2 (Nrf2), signal transducers and activators of transcription (STAT), and mitogen-activated protein kinase (MAPK), and are capable of influencing gene expression. Furthermore, they exhibit anti-inflammatory effects by inhibiting nuclear factor kappa B (NF-κB) activation and suppressing the expression of pro-inflammatory cytokines and chemokines [9].

Despite their beneficial effects, the therapeutic potential of polyphenols is significantly limited by their poor bioavailability. Two primary barriers restrict their efficacy: first, polyphenols administered orally undergo extensive metabolism in the gastrointestinal tract and exhibit low permeability across the gut epithelium; second, even if absorbed into the bloodstream, polyphenols must cross the blood–brain barrier (BBB) to reach the central nervous system (CNS). Although some polyphenols are capable of penetrating the BBB, their CNS concentrations remain suboptimal [10,11]. The overall understanding of polyphenol biodistribution in the CNS remains limited. Challenges include poor bioavailability, complex interactions with proteins and receptors, and only partially informative in vitro data. These limitations, combined with the invasive nature of sampling cerebrospinal fluid (CSF) or brain tissue in humans, contribute to limited direct clinical evidence on polyphenol BBB penetration and activity in the human CNS. Most current data are derived from animal models and in vitro studies [11,12]. Nonetheless, a few studies have directly assessed BBB penetration of polyphenols. Detection was performed using CSF or brain tissue samples. Following oral administration, polyphenols such as caffeic acid, homovanillic acid, 3-hydroxyphenylacetic acid, resveratrol, and its derivatives were detected in the CNS [13,14]. In contrast, curcumin was not detected in CNS tissues, despite extensive research into its therapeutic effects. Some studies have reported curcumin’s potent antioxidant properties, ability to inhibit protein misfolding and aggregation, and ability to alleviate Parkinsonian symptoms and reduce migraine severity. However, other clinical data have shown no significant effect of curcumin in Alzheimer’s patients, possibly due to its inability to cross the BBB in effective concentrations [12,15]. There are also limited clinical studies supporting the health-promoting effects of polyphenols. Gu et al. [16] demonstrated that resveratrol administered at a dose of 500 mg/day over 52 weeks resulted in decreased levels of Aβ40 in CSF and plasma compared to placebo. Additionally, participants in the treatment group experienced less motor function decline and a decrease in brain volume [16]. Another population-based study involving 1572 adults living in Italy reported that the moderate consumption of coffee and tea (rich in chlorogenic acids and catechins, respectively) was associated with lower perceived stress. Moreover, individuals who regularly consumed coffee and moderate to regular amounts of red wine (a source of resveratrol) exhibited fewer depressive symptoms. However, no significant effects on sleep quality were observed. The authors emphasized the need for more precise and controlled studies [17]. In a further clinical meta-analysis, researchers examined the influence of polyphenol supplementation on the expression of brain-derived neurotrophic factor (BDNF), a key regulator of neuronal development and function. The findings revealed a significant increase in BDNF levels, which correlated with improved cognitive performance and reduced mental fatigue. The authors noted that the effects were dose- and regimen-dependent, with more pronounced benefits observed after acute supplementation [18]. An interesting review was presented by Hunt et al. [19]. The authors compiled a list of human studies in which the intervention involved polyphenol intake through the diet, followed by an evaluation of the resulting effects. Most of the studies included in the review were conducted on healthy individuals (20 out of 28). Interestingly, only 6 out of the 28 studies reported no observable effects. The effects of polyphenols were most commonly associated with improvements in mood and reductions in mental fatigue, as well as neuroprotection and support of cognitive functions. One example from a study involving patients with Alzheimer’s disease showed that administration of *Ginkgo biloba* extract at a dose of 120 mg/day for six months led to a significant improvement in the mini-mental state examination score [20]. Resveratrol is a polyphenol whose blood concentration increases linearly with the administered dose [21]. However, the bioavailability of most polyphenols does not follow this pattern. When analyzing the literature on polyphenols, one may get the misleading impression that increasing the dose automatically enhances clinical outcomes. In reality, the nature of polyphenol bioavailability is far more complex. It is essential to consider the metabolic fate of polyphenols and their distribution within the body [22]. For instance, a polyphenol that is rapidly metabolized by the gut microbiota may never reach the bloodstream in its unaltered form. Furthermore, the resulting metabolites may exhibit a different range of biological activities compared to the parent compound [23]. In the context of CNS disorders, the BBB represents an additional variable that some polyphenols are unable to cross [10]. Therefore, the clinical efficacy of a polyphenol depends not only on the amount absorbed into the bloodstream but also on its ability to reach the site of action and the chemical form in which it arrives.

To overcome the bioavailability limitations of polyphenols, considerable attention has been directed toward nanodelivery platforms, particularly liposomes. Liposomes are spherical nanocarriers composed of phospholipid bilayers capable of encapsulating both hydrophilic and hydrophobic compounds. Several liposomal drug formulations, especially in oncology, have already received approval for clinical use. Due to their biocompatibility, modifiability, and ability to cross biological barriers, liposomes represent a promising vehicle for polyphenol delivery. Nonetheless, data specifically addressing liposome-mediated polyphenol delivery to the CNS remain limited.

The aim of this review is to provide an overview of the mechanisms by which selected polyphenols exert neuroprotective effects in the context of neurodegenerative and neuroinflammatory disorders. Special emphasis is placed on the activity of individual polyphenols in distinct CNS-resident cell types. Furthermore, this review highlights the critical issue of polyphenol bioavailability and introduces liposomes as a promising nanocarrier system. Selected literature is presented to illustrate the potential of liposomes as a nanodelivery platform for overcoming current limitations in polyphenol-based therapy.

## 2. Polyphenols as Neuroprotective Agents: Antioxidant Properties, Bioavailability, and Mechanisms of Action

Polyphenols are well-known antioxidant compounds, and their consumption is associated with positive effects on health. They scavenge ROS such as superoxide anion (O_2_^•−^)), hydroxyl radical (·OH), and hydrogen peroxide (H_2_O_2_), thereby silencing inflammation and preventing and/or moderating the course of diabetes, cardiovascular disease, cancer, and other diseases associated with oxidative damage [24,25]. Three major mechanisms underlie the antioxidant activity of polyphenols: hydrogen-atom transfer (HAT), single-electron transfer (SET), and transition-metal chelation (TMC) [26]. The HAT mechanism is based on breaking the bond between oxygen and hydrogen (O-H) in order to transfer the hydrogen atom to a free radical and inactivate it [27]. For the SET mechanism, polyphenols play the role of electron donors to free radicals, whereas in the TMC mechanism, they act anti-oxidatively by chelating with a metal ion and forming a stable complex [28]. These properties place polyphenols as an unquestionable factor contributing to health maintenance. Another antioxidant mechanism involves an interaction with receptors or enzymes participating in signal transduction. Moreover, polyphenols influence detoxification systems such as glutathione peroxidase (GPx), superoxide dismutase (SOD), or catalase (CAT). Polyphenols cause an increase in activity of these enzymes, contributing to more efficient detoxification [29]. Interestingly, polyphenols can also act as co-antioxidants, participating in the regeneration of essential vitamins. An example of this mechanism is the synergistic antioxidant activity observed between α-tocopherol and polyphenols derived from green tea. During the antioxidant action of α-tocopherol, its radicals are produced, which can then be regenerated by a co-antioxidant, such as vitamin C or green tea polyphenols. The regeneration significantly enhances the antioxidant properties of α-tocopherol while also eliminating the so-called tocopherol-mediated peroxidation [30]. Nevertheless, several studies have questioned the direct antioxidant effects of polyphenols in humans. In vivo studies investigating the antioxidant activity of flavonoids in humans have yielded inconsistent results, showing antioxidant effects, no significant impact, or even mild pro-oxidant effects [31]. Although polyphenols are typically described as potent antioxidants, their oxidized forms can contribute to the production of O_2_^•−^, H_2_O_2_, and complex mixtures of semiquinones and quinones, which may be cytotoxic [31]. It is also important to note that much of the current understanding of the mechanisms of polyphenol action is based on cell culture models, which have significant limitations and may not accurately reflect physiological conditions. In the context of studying the antioxidant properties of polyphenols, one of the main confounding factors is the oxygen concentration, which, under standard in vitro conditions, can be 15 to 150 times higher than that found in human tissues. This persistent oxidative stress may alter cellular responses and distort experimental outcomes [32,33,34]. A list of studies with such artifacts is presented in Halliwell’s review [31]. Although the properties of polyphenols as a direct antioxidant in vivo have been questioned, it should not be overlooked that they are increasingly recognized as bioactive compounds with pleiotropic effects. These include antibacterial, immunomodulatory, anti-inflammatory, genoprotective, and anti-cancer properties. Given the focus of this work, the most compelling aspect of polyphenol activity appears to be their involvement in the regulation of inflammation. It has been demonstrated that polyphenols can modulate gene expression, suppress the production of pro-inflammatory cytokines, and influence the activity of immune cells [35]. Furthermore, when administered orally, polyphenols exert a significant impact on the composition and function of the gut microbiota. Some polyphenols have been shown to beneficially alter microbial diversity, promote the growth of bacteria that produce short-chain fatty acids, and modulate the synthesis of neurotransmitters and hormones [36].

### 2.1. Bioavailability of Polyphenols and Their Biosafety

In the health context, the biggest challenge regarding polyphenols is their bioavailability. Polyphenols represent a wide and chemically diverse group of bio-compounds, and, thus, it can be assumed that their bioavailability is not homogeneous. Curcumin, resveratrol, and quercetin, when administered orally, are absorbed at a range lower than 1%, although the glycoside form of quercetin can achieve absorption rates exceeding 17% [37,38,39]. Anthocyanidins are absorbed at a rate of 1–2% [40]. The absorption of flavan-3-ols is dependent on the source; for example, green tea consumption results in 2–15% absorption, while the consumption of cocoa leads to 5–10% absorption [41,42]. In contrast, isoflavones and hydroxytyrosol are examples of polyphenols with relatively high bioavailability [43,44]. Polyphenols administered orally are exposed to numerous factors that affect their bioavailability. First, polyphenols from natural sources may be embedded in a matrix or form part of a phytocomplex with other molecules, complicating the prediction of their absorption. Second, physical and chemical properties, as well as the entire digestive process, from mixing with saliva to intestinal absorption, along with the stability of the digestion process and the impact of gut microbiota, are crucial in determining the final concentration of unmodified polyphenols in plasma [45]. When considering food-derived polyphenols, the influence of the food preparation process itself should not be overlooked. Some polyphenols, such as quercetin, flavonols, and other glycosidic polyphenols, may exhibit increased absorption in the presence of glucose, facilitated by sodium-dependent glucose transporter 1 (SGLT1), which can transport these polyphenols [46]. Meanwhile, resveratrol, known for its positive biological effects on human health, is metabolized very rapidly, primarily through two reactions: sulfation and glucuronidation, which occur in the gut and liver [47].

The intake of polyphenols from food is associated with the influence of the food matrix on the bioavailability of these polyphenols. It can promote absorption or reduce it. In the example of flavonoids, such enhancers of absorption can be oils, lipids, or vitamins, while minerals or dietary fibers reduce this process [48]. Another noteworthy fact is the ability of certain natural compounds, including polyphenols, to reduce the activity of enzymes responsible for polyphenol metabolism. One example is quercetin, which decreases the activity of glucuronidation and sulfation processes, the primary metabolic pathways for resveratrol. When quercetin and resveratrol are administered together, the bioavailability of resveratrol increases significantly [49]. Another bioavailability enhancer is piperine, an alkaloid that, in in vitro studies, has been shown to reduce the metabolism of resveratrol. However, there is no consensus among researchers regarding the effect of piperine on the final plasma concentration of resveratrol. Animal model studies support the findings from in vitro research, but clinical trials have not demonstrated a significant increase in plasma resveratrol levels when co-administered with piperine [50,51].

The consumption of polyphenols from natural and pharmaceutical products is considered generally safe. Their safety has been confirmed over the short, medium, and long terms [52]. However, these effects are dose-dependent. Amiot et al. [53] investigated the effects of polyphenols on features of metabolic syndrome in humans. Based on a series of clinical studies reviewed in their paper, it can be observed that strong evidence supporting the breakthrough efficacy of polyphenols is lacking. However, their beneficial effects appeared more pronounced at higher concentrations. The maximum doses of polyphenols used in the studies cited in the review generally did not exceed 1000 mg [53]. There is also evidence that high concentrations of polyphenols may exert toxic effects [7,54]. Nevertheless, both the optimal and toxic doses should be established individually for each polyphenol. For instance, curcumin administered at 2000–5000 mg/kg did not cause adverse effects [55]. Resveratrol and quercetin were found to be non-toxic at concentrations of 300 μg/mL and 2.3–6 g/kg, respectively. However, doses exceeding 1000 mg/kg for resveratrol and 8 g/kg for quercetin were associated with toxicity [56,57]. Table 1 presents selected examples of adverse effects that polyphenols may cause, along with the corresponding doses at which these effects were observed in the reviewed studies. As previously mentioned, the consumption of polyphenols through diet and supplements is generally considered safe. However, their beneficial effects often emerge only at doses significantly higher than those achievable from natural sources. Therefore, efforts to improve polyphenol bioavailability must also account for their potential side effects, including the potential accumulation of polyphenols in tissues or their metabolites produced by the gut microbiota. It is important to note that much of the current scientific knowledge regarding polyphenols is based on in vitro studies, which do not always accurately reflect in vivo responses in humans. Future research should prioritize the generation of robust clinical data, the identification of optimal dosing strategies, and a deeper understanding of interactions between polyphenols, the microbiota, proteins, and cellular structures, as well as the identification of patient subgroups particularly vulnerable to adverse effects.

### 2.2. Neuroprotective Mechanisms of Polyphenols’ Action in the Context of Oxidative Stress and Neurodegeneration

Oxidative stress is a natural consequence of oxidative respiration. In physiological conditions, cells are equipped with numerous mechanisms to counteract the negative effects of ROS, thereby avoiding serious damage. However, oxidative stress appears to be one of the probable causes of neuropathological states, as observed in neurodegenerative diseases associated with age, such as AD, PD, and Huntington’s disease (HD) [67]. A long-term decrease in the antioxidative capacities of cells has been observed in these disorders.

**Figure 2:**
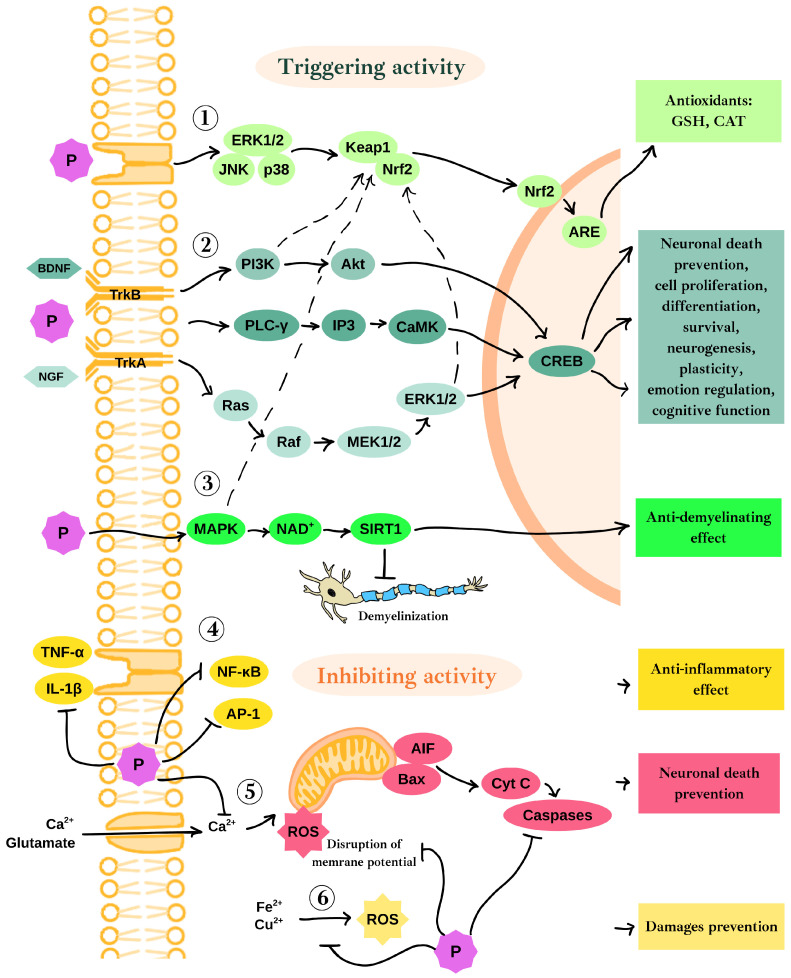
Triggering (**1**–**3**) and inhibiting (**4**–**6**) activity of polyphenols in brain cells’ signaling pathways in neurodegenerative context. “P” is the symbol of polyphenol. CAT and GSH expression are upregulated owing to the activation of Keap1/Nrf2/ARE pathway by polyphenols, which results in enhanced antioxidative effect (**label 1**). Directly, polyphenols have an impact on TrkA and TrkB receptors, leading to neuroprotection and regulation of cell proliferation, differentiation, and plasticity through PI3K/Akt, PLC-γ, and MAPK/ERK pathway activation (**label 2**). Moreover, polyphenols activate SIRT1 and attenuate demyelination (**label 3**). They inhibit the release of proinflammatory cytokines as well as NF-κB and AP-1 expression (**label 4**). Additionally, they reduce neuronal death due to mitochondrial membrane potential disruption, affecting the calcium ion influx (**label 5**). They prevent cellular damage by suppressing ROS formation induced by iron and copper ions (**label 6**). Abbreviations: AIF—apoptosis inducing factor; Akt—protein kinase B; AP-1—activator protein 1; ARE—antioxidant response element; Bax—Bcl-2-associated X protein; BDNF—brain-derived neurotrophic factor; CaMK—Ca^2+^/calmodulin-dependent protein kinase; CAT—catalase; CREB—cyclic response element binding protein; Cyt C—cytochrome c; ERK1/2—extracellular signal-regulated kinases 1/2; GSH—glutathione; IL-1β—interleukin 1 beta; IP3—inositol trisphosphate; JNK—c-Jun N-terminal kinase; Keap1—Kelch-like ECH-associated protein; MAPK—mitogen-activated protein kinases; MEK1/2—mitogen-activated protein kinase kinase 1/2; NAD+—nicotinamide adenine dinucleotide; NF-κB—nuclear factor kappa-light-chain-enhancer of activated B cells; NGF—nerve growth factor; Nrf2—nuclear factor erythroid 2-related factor 2; PI3K—phosphoinositide 3-kinase; PLC-γ—phospholipase C-gamma; ROS—reactive oxygen species; SIRT1—silence information regulator 1; TNF-α—tumor necrosis factor alpha; TrkA—tropomyosin receptor kinase A; TrkB—tropomyosin receptor kinase B.

Polyphenols exhibit neuroprotective effects in various ways. Figure 2 presents a graphical summary of the neuroprotective mechanisms of polyphenols. One of them is the activation of the main protective pathway against endogenous and exogenous ROS, namely the Kelch-like ECH-associated protein 1 (Keap1)/Nrf2/antioxidant responsive element (ARE) pathway [68]. Nrf2 is a transcription factor that triggers the expression of genes responsible for maintaining redox status in neuronal cells and protecting against the effects of oxidative stress. Under physiological conditions, Nrf2 is bound to Keap1 in the cytoplasm. Oxidative damage or interactions of polyphenols with receptor kinases, extracellular signal-regulated kinase 1 and 2 (ERK1/2), c-Jun N-terminal kinase (JNK), and p38 protein cause the release of Nrf2, which then translocates to the nucleus, where it couples to ARE, leading to expression of antioxidants such as CAT and glutathione (GSH) (label 1 in Figure 2) [69]. Moreover, a number of upstream signaling pathways such as MAPK, phosphatidylinositol-3-kinase (PI3K)/Akt, and ERK are capable of activating Nrf2 independently or in combination [70]. Polyphenols also exhibit anti-neuroinflammatory properties by preventing the release of cytokines, including interleukin-1β (IL-1β) and tumor necrosis factor α (TNF-α), as well as by suppressing the expression of NF-κB and activator protein 1 (AP-1) (label 4 in Figure 2). Additionally, the MAPK pathway, regulated by TNF-α, plays a role in reducing the production of proinflammatory cytokines. These mechanisms collectively contribute to the overall anti-inflammatory effects of polyphenols in the nervous system [9].

Polyphenols may cause the activation of two membrane receptors crucial for neuronal development and biology: tropomyosin kinase B (TrkB) and tropomyosin kinase A (TrkA). TrkB receptor serves as a specific receptor for BDNF, while TrkA functions as the receptor for nerve growth factor (NGF); both are widely expressed in the mammalian brain. BDNF and NGF, along with their respective receptors and the glial cell line-derived neurotrophic factor (GDNF), are crucial for processes such as adult synaptic plasticity, memory formation, neurite extension, neurotrophic support, and the activation of pathways that protect neurons [71]. When BDNF or polyphenols stimulate TrkB, they trigger three significant intracellular signaling pathways: PI3K/Akt, phospholipase C-γ (PLC-γ), and MAPK/ERK (label 2 in Figure 2). These pathways eventually result in the phosphorylation of the cAMP response element-binding protein (CREB), which in turn regulates gene transcription in neurons. CREB, a transcription factor predominantly found in neurons, is abundant in the brain and plays a critical role in learning and memory [72]. The survival of neurons is closely associated with the levels of phosphorylated CREB (pCREB). In the hippocampus, the activation of PI3K and its downstream target Akt has been shown to increase CREB phosphorylation, through which the death of neurons is prevented. The ERK pathway, part of the broader MAPK family, plays a vital role in several neuronal functions, including cell proliferation, differentiation, and survival by promoting the expression of survival genes and inhibiting proteins that lead to apoptosis. CREB, as a downstream effector of the ERK pathway, is implicated in neurogenesis, plasticity, emotional regulation, and cognitive functions [73]. Furthermore, the excessive activation of glutamate receptors leads to a significant influx of calcium, which disrupts the mitochondrial membrane potential (label 5 in Figure 2). This disruption results in the increased production of ROS and induces apoptosis in various brain diseases, driven by elevated levels of B-cell leukemia/lymphoma 2 (Bcl-2)–associated X protein (Bax), the release of cytochrome C, and the activation of caspases [74]. Additionally, apoptosis-inducing factor (AIF) is cleaved and released from mitochondria into the cytosol and subsequently into the nucleus in the presence of increased Ca^2+^ ion levels, leading to numerous consequences. In the nucleus, AIF initiates the fragmentation of DNA, chromatin condensation, and caspase-independent cell death; moreover, it is involved in free-radical generation and energy loss [75]. Elevated levels of Bax further drive the apoptotic process by incorporating it into the mitochondrial outer membrane, leading to the release of cytochrome C and AIF into the cytosol. Polyphenols protect mitochondria against the depolarization of their membrane, mediated by glutamate receptor activation. In consequence, polyphenols help protect neurons from mitochondria-dependent apoptotic death, AIF-dependent apoptosis, and the release of caspases [74].

The frequent consumption of polyphenols contributes to a reduced risk of stroke and its severity by decreasing the activity of matrix metalloproteinases (MMPs), regulating lipid peroxidation in neurons, and controlling dysregulated Ca^2+^ levels [74,76]. Additionally, polyphenols may also show an anti-demyelinating effect by sirtuin 1 (SIRT1) activation, which positions them as potential therapeutic molecules for demyelinating diseases (label 3 in Figure 2). Copper and iron ions, along with their accumulation in the CNS (label 6 in Figure 2), play a significant role in the formation of ROS and the pathogenesis of neurodegenerative diseases such as multiple sclerosis (MS), AD, PD, and HD [77,78]. On the other hand, polyphenols possess strong chelating properties, thus preventing damage caused by metal-associated ROS, inflammation, and neurotoxicity. Moreover, polyphenols are involved in neutralizing misfolded tau proteins and inhibiting acetylcholinesterase activity, thereby offering protection against the development of AD and dementia [9].

## 3. CNS Cell-Type Specific Effects of Polyphenols in the Central Nervous System

**Figure 3:**
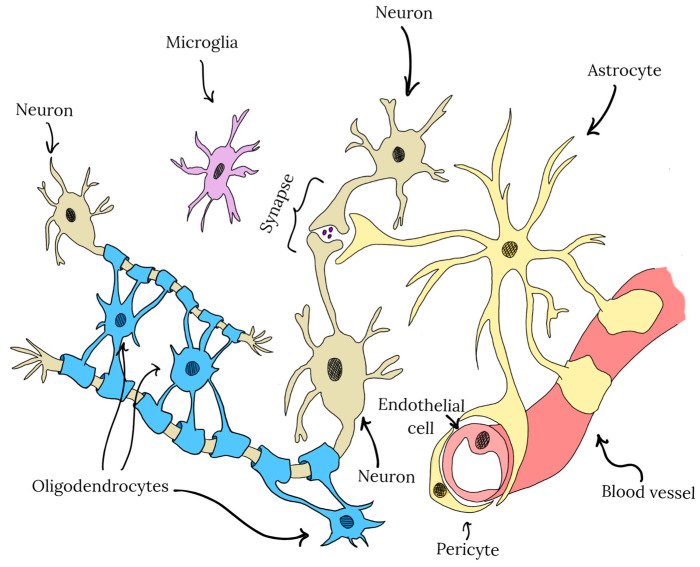
Cellular composition in CNS.

CNS is composed not only of neurons but also of a wide range of non-neuronal cells. The major cell types are illustrated in Figure 3. In addition to neurons, which are primarily responsible for signal transmission, the CNS comprises various types of glial cells, including astrocytes, microglia, and oligodendrocytes, which are crucial for neuronal survival, function, and the maintenance of internal CNS homeostasis [79].

Due to their diverse roles and varying sensitivities to oxidative stress, polyphenols exert cell-type-specific effects within the CNS. Neurons, which are particularly vulnerable to oxidative damage, benefit from polyphenols through the enhancement of antioxidant defenses and reduction in ROS levels and metal ion accumulation. In astrocytes, polyphenols exhibit modulating properties of key homeostatic functions, including the production of cytokines, neurotrophic factors, and the regulation of neurotransmitter metabolism. Microglia, the primary immune cells of the CNS involved in neuroinflammatory responses, exhibit reduced reactivity in the presence of polyphenols, which may mitigate the detrimental effects of chronic inflammation. In contrast, relatively little is known about the impact of polyphenols on oligodendrocytes, the cells responsible for neuronal myelination. However, existing studies suggest that polyphenols may support oligodendrocyte differentiation and promote myelin regeneration [79]. Another critical element of the CNS microenvironment is the BBB, composed of neurovascular endothelial cells, astrocytes, and pericytes. The BBB plays a central role in maintaining CNS homeostasis by forming a highly selective barrier that creates a sterile and stable environment distinct from the peripheral circulation. In the context of polyphenol delivery to the CNS, the BBB represents a major obstacle, as it significantly limits the penetration of polyphenols into the CNS [80]. Considering the cellular diversity and structural complexity of the CNS, a thorough understanding of how individual polyphenols interact with specific CNS cell types is essential. This knowledge is critical not only for elucidating the neuroprotective and therapeutic mechanisms of polyphenols but also for assessing potential adverse effects associated with their use.

### 3.1. Neuroprotective Mechanisms of Selected Polyphenols in Neuronal Cells

Neurons are the primary functional cells of the CNS. Their structure includes a cell body and processes such as dendrites and axons, through which nerve impulses are received and transmitted. Importantly, neurons are non-dividing cells and possess relatively low antioxidant capacity compared to other brain cells. This is mainly due to the weak activation of the Nrf2 pathway. Under physiological conditions, neurons exhibit high and variable mitochondrial activity, which generates significant amounts of ROS. This oxidative stress is counterbalanced by astrocytes, maintaining homeostasis and preventing damage [81]. Thus, in the context of chronic or intense inflammation, neurons are highly susceptible to stress, which can ultimately lead to their death. In the early stages of neurodegenerative diseases, neuronal loss may be partially compensated by the plasticity of remaining neurons. However, when the extent of loss surpasses the brain’s capacity for compensation, cognitive and motor deficits emerge [82]. Neurodegenerative and neuroinflammatory disorders differ in their etiology and course, yet all are characterized by progressive neuronal loss. They commonly involve inflammation, oxidative stress, and mitochondrial dysfunction. Neuronal death is caused by multiple factors; however, certain pathways are shared among AD, PD, and MS. These include PI3K/Akt and Janus kinase (JAK)/STAT, which regulate neuronal survival and responses to oxidative stress, as well as the C-X-C motif chemokine 12 (CXCL12)/C-X-C chemokine receptor type 4 (CXCR4)/C-X-C chemokine receptor type 7 (CXCR7) axis, strongly associated with cognitive decline and motor neuron degeneration. This signaling network interacts with PI3K/Akt, JAK/STAT, and MAPK pathways. Furthermore, the excessive accumulation of metal ions, such as zinc, copper, manganese, and iron, contributes to neurodegeneration by promoting oxidative stress [83,84,85].

#### 3.1.1. Neuroprotective Effects of Epigallocatechin-3-Gallate in Neurons

Epigallocatechin-3-gallate (EGCG) is the major polyphenol found in green tea leaves, known for its potent antioxidant properties. In the context of neurodegenerative and neuroinflammatory diseases, EGCG exhibits a wide range of protective effects on CNS cells. In neurons, EGCG confers neuroprotection through multiple mechanisms, including the modulation of intracellular signaling pathways, the suppression of inflammation, the mitigation of oxidative stress, and the prevention of amyloid beta (Aβ) aggregation. EGCG influences several key signaling cascades involved in neuronal survival and oxidative defense. For instance, it has been shown to activate the MAPK and PI3K/Akt pathways, as well as pathways associated with calcium ion influx, thereby enhancing neuronal viability [86]. In an in vivo study, EGCG significantly mitigated sevoflurane-induced neurodegenerative changes and activated the CREB/BDNF/TrkB-PI3K/Akt signaling axis, resulting in improved memory and learning performance in mice [87]. Similarly, in a murine model of Parkinson’s disease, EGCG was found to reduce the expression of mTOR, Akt, and glycogen synthase kinase-3 beta (GSK-3β) and inhibit neuronal apoptosis [88]. EGCG also plays a direct role in ROS neutralization, owing to its B-ring structure and biological activity. It upregulates antioxidant enzymes such as SOD and CAT, enhancing the cell’s antioxidant defense [89,90]. In the context of AD, EGCG demonstrates a multifaceted mechanism of action against Aβ aggregation. It inhibits Aβ aggregation, reduces cytotoxicity in Neuro-2a neuronal cells, and ameliorates Aβ-induced memory impairment in mice [91,92]. Furthermore, EGCG has been shown to regulate iron accumulation and lower intracellular calcium levels, which supports neuronal survival and functional integrity [91].

#### 3.1.2. Neuroprotective Effects of Berberine in Neurons

Berberine is a well-known polyphenol belonging to the group of alkaloids with broad biological activity. Similar to other polyphenols, it modulates several key signaling pathways involved in antioxidant defense, apoptosis regulation, and neuronal survival. Under specific pathological conditions of neurodegenerative diseases, berberine has also demonstrated anti-inflammatory, anti-amyloidogenic, and neuroprotective properties, improving CNS function in in vivo studies [93]. Neurons in neurodegenerative diseases are particularly susceptible to apoptosis. Berberine influences this process in a dual manner: it promotes the expression of anti-apoptotic proteins such as Bcl-2 and regulates autophagy-related proteins, thereby improving the Bax/Bcl-2 ratio, while simultaneously downregulating pro-apoptotic factors including cytochrome c, Bax, and caspases [93,94]. Berberine also mitigates ROS accumulation by activating the PI3K/Akt/Nrf2 signaling cascade [95]. Several studies confirm its protective effects in neuronal models. In SH-SY5Y cells exposed to glucose-induced toxicity, berberine upregulated heme oxygenase 1 (HO-1) and NGF expression through Nrf2 activation and modulated the insulin-like growth factor 1 (IGF-1)/Akt/GSK-3β pathway under both physiological and hyperglycemic conditions [96,97]. In Alzheimer’s disease models, berberine has shown promise by reducing both intracellular and extracellular Aβ levels, modulating key proteins involved in amyloid metabolism, such as amyloid precursor protein (APP) and β-secretase 1. These changes promoted autophagy and limited Aβ aggregation [98].

In a 6-hydroxydopamine model of Parkinson’s disease, berberine treatment was associated with increased HO-1 expression, reduced ROS levels, and a decrease in neuronal death [99]. However, in addition to its neuroprotective effects, berberine may also exert harmful or negligible effects, particularly at higher concentrations. While low concentrations have been shown to be neuroprotective, higher doses may exert neurotoxic effects. One study reported that a low dose provided neuroprotection, whereas a higher dose failed to produce the same benefit [100]. Another investigation demonstrated mitochondrial dysfunction at concentrations exceeding 1 μM [101]. Furthermore, additional reports have described adverse outcomes, including dopaminergic neuron loss in the substantia nigra following berberine administration [102,103].

#### 3.1.3. Neuroprotective Effects of Curcumin in Neurons

Curcumin is a well-known polyphenol, and numerous studies confirm its beneficial properties, particularly as an anti-diabetic, anti-cancer, immunomodulatory, antioxidant, and anti-inflammatory molecule [104]. In the context of neurodegenerative diseases, curcumin acts through anti-inflammatory, antioxidant, and neuroprotective mechanisms by modulating signaling pathways, scavenging ROS, and preventing the production of pathological proteins [95]. A study considering the effect of curcumin on diaminobutyric acid-induced cytotoxicity showed that it inhibits NF-κB, cyclooxygenase-2 (COX-2), inducible nitric oxide synthase (iNOS), TNF-α, complement components, proinflammatory cytokines, nod-like receptor (NLR) family pyrin domain containing 3 (NLRP3), and ROS and promotes Nrf2 and interleukin-10 (IL-10), thereby exerting neuroprotective effects [105]. Interestingly, curcumin derivatives have been shown to modulate multiple signaling pathways, like the Akt/mTOR pathway, the NF-κB pathway, the Nrf2, β-catenin pathway, the BDNF/TrkB pathway, and the Interleukin-6 (IL-6)/STAT3 axis, all of which are associated with oxidative stress, inflammatory processes, and neuronal survival. Moreover, an experiment was performed in which, among 214 compounds, curcumin showed the highest affinity for Aβ; thus, it may be considered a factor reducing AD pathogenesis [106]. Furthermore, in studies using Aβ oligomer-induced neurotoxicity, curcumin demonstrated broad anti-inflammatory and neuroprotective activity through the suppression of proinflammatory cytokines, the regulation of SIRT1, SOD, and GSH, and a reduction in ROS and Aβ aggregation [107,108,109]. Indeed, numerous studies confirm its significant role not only in the context of AD but also in PD. For instance, curcumin improves neuronal survival by regulating metabolism and activating sirtuin 3, which was also confirmed in an in vivo study [110]. In PD models, curcumin exhibits neuroprotective effects by downregulating p53 and the Bax/Bcl-2 ratio, both associated with apoptosis [111]. It also reduces aggregation, increases the solubility of α-synuclein, and prevents neuronal death, while increasing resistance to ROS and inflammation [106,112]. Additionally, curcumin contributes to reducing iron- and aluminum-dependent damage [113,114].

#### 3.1.4. Neuroprotective Effects of Resveratrol in Neurons

Resveratrol, a member of the stilbene family, found in grapes and red wine, has long attracted the interest of researchers. One of the first studies involving this compound was published in 1993, in which Frankel et al. demonstrated that trans-resveratrol, when added to human low-density lipoprotein, reduced copper-catalyzed oxidation [115]. The main research areas related to resveratrol include cardiovascular diseases, cancer, metabolic disorders, and CNS disorders [116]. Compared to curcumin, berberine, and EGCG, resveratrol appears to have the least direct impact on neurons. Nevertheless, it is attributed with a neuroprotective role, partly by activating the PI3K/Akt/mTOR pathway and inhibiting NF-κB, which leads to reduced inflammation and suppressed apoptosis [117,118]. Other potential antioxidant mechanisms include triggering mitochondrial biogenesis via the activation of 5′AMP-activated protein kinase (AMPK) or activating the peroxisome proliferator-activated receptor (PPAR) and promoting the deacetylation of peroxisome proliferator-activated receptor-gamma coactivator 1-alpha (PGC-1α) [119,120]. Moreover, resveratrol may exert neuroprotective effects by activating ERK-induced CREB signaling, which in turn modulates several neurotrophic and immunomodulatory factors, such as BDNF and NGF [121]. The most significant neuroprotective activity of resveratrol appears to be its activation of SIRT1. In neurons, SIRT1 plays a key role in regulating excitability, synaptic plasticity, development, and protection against oxidative stress and neurodegeneration. This activity contributes to supporting mitochondrial function, suppressing inflammation, and promoting neuronal survival. In studies assessing resveratrol activity across various contexts, SIRT1 activation is frequently reported, along with associated neuroprotective and anti-apoptotic effects [121]. In PD, SIRT1 activation may enhance the autophagic degradation of α-synuclein [119]. Additionally, resveratrol may regulate the expression of Bax and Bcl-2 proteins, thereby promoting anti-apoptotic effects, and protect neurons from oxidative stress by acting as a scavenger [122]. In AD models, resveratrol has been shown to reduce iNOS expression, upregulate HO-1, and thereby alleviate oxidative damage and mitigate Aβ-induced neurotoxicity [123].

#### 3.1.5. Neuroprotective Effects of Quercetin in Neurons

Quercetin is a flavonoid that has been extensively studied across various areas of human health. In the context of neurodegenerative diseases, it acts primarily by counteracting oxidative stress, inhibiting neuroinflammation, and regulating key signaling pathways. While discussing the impact of quercetin on neurons, it is important to note that it may exert adverse effects at higher concentrations. There have been reports of anti-proliferative, apoptotic, and genotoxic effects of quercetin at doses above 100 μM in certain cell types [124]. In neurons, quercetin functions similarly to other polyphenols by modulating signaling pathways associated with oxidative stress response, regulating apoptosis, and acting as a neuroprotective and antioxidant agent. There are numerous studies highlighting quercetin’s influence on neuronal adaptation, synaptic plasticity, and learning. Reported neuroprotective pathways include PI3K/AKT/Nrf2, SIRT1, and ERK/CREB/BDNF [125]. Additionally, quercetin has been shown to activate the Wnt, p38, and MAPK signaling pathways, which contribute to enhanced neuronal viability by regulating cell death, proliferation, neuroplasticity, and the response to oxidative stress [126]. Quercetin’s antioxidant action is not limited to direct free radical scavenging. It also inhibits xanthine oxidase and nitric oxide synthase and activates the Nrf2/ARE pathway [127]. There is also evidence that quercetin can prevent neuronal damage caused by hyperoxide, H_2_O_2_, or neurotoxic agents like Aβ and 6-hydroxydopamine [126]. In the context of PD, quercetin provides neuroprotection by suppressing excessive nitric oxide production and downregulating iNOS and pro-inflammatory genes, such as IL-1β and TNF-α [128]. Furthermore, quercetin has been shown to protect neurons from Aβ oligomer-induced cytotoxicity, inhibit Aβ aggregation, reduce protein and lipid oxidation, and prevent apoptosis. This positions quercetin as a promising molecule for improving AD outcomes [129]. Quercetin is also involved in reducing intracellular ROS levels and glutamate-induced Ca^2+^ influx [130]. In addition, quercetin improves cognitive performance in animal models, which has been linked to a reduction in acetylcholinesterase activity [131].

### 3.2. Neuroprotective Mechanisms of Selected Polyphenols in Astrocytes

Astrocytes are the most abundant cells in the CNS. With their cytoplasmatic processes, astrocytes ensheathe synapses, make contact with brain capillaries, and sense and rapidly react to any changes in the extracellular environment [132]. Numerous findings describe their role in the modulation of synapse signal transduction and neuron activation in multiple ways [133]. In fact, astrocytes, as an important source of BDNF, provide neurotrophic support for neurons, regulating not only neuron survival but also their complex activity and memory formation [134]. In addition, astrocytes support neurons in the formation of synapses and electric signal transduction, provide ion homeostasis, regulate synapse biology, and modulate neuron excitation as a part of tripartite synapses [135,136]. What is more, astrocytes take part in CNS immune response as a source of inflammation-regulating agents: chemokines, cytokines, and extracellular vesicles [137,138]. Astrocytes are also a functional part of the BBB, where their secretory activity regulates the biology of brain endothelium and BBB tightness [139].

Astrocytes, as key regulators of CNS cellular activity, may be considered an important target for polyphenols. It was found that dietary polyphenols may modulate the astrocytic activity and regulate their functions, contributing to changes in brain activity. Numerous studies have reported the beneficial impact of particular polyphenolic compounds on astrocyte activity and their impact on neural tissue biology. Their activity may be related to the attenuation of astrocytes’ inflammatory potential. Polyphenol-rich extracts from *Limonium gmelina* were reported to reduce oxidative stress in astrocytes exposed to H_2_O_2_, as well as in pro-inflammatory conditions provided by TNF-α [140]. Such activity may minimize brain tissue destruction during inflammation. What is more, pomegranate polyphenolic extracts were reported to upregulate IL-10 expression in human astrocytes (U373-MG cells) exposed to lipopolysaccharide (LPS), which may suggest protective activity in systemic inflammation characteristic of AD development. However, the effect on protein level was not measured [141]. In another study, quercetin and phytochemical extracts were shown to reduce the LPS-driven NF-κB-controlled inflammatory response and activate the Nrf2/ARE response in rat astrocytes [142]. Similar activity was observed for other studied polyphenols, including cyanidin and cyanidin-3-O-glucoside, although high doses were required. Comparable effects were also reported for botanical extracts derived from *Withania somnifera*, *Sutherlandia frutescens*, and *Euterpe oleracea*. Furthermore, the flavonoid epicatechin was shown to activate the Nrf2/ARE pathway through polyphenol-dependent PI3K activation [143]. An inhibitory effect on the STAT3/NF-κB signaling pathway in astrocytes was reported for lignan e*nt*-Sauchinone, where the reduction in ROS level was observed in cells exposed to this polyphenol. Moreover, in this study, the authors described the ability of e*nt*-Sauchinone to inhibit LPS-induced Aβ_1–42_ secretion, suggesting its potential to reduce amyloid-β deposition [144]. Generally, this may suggest a protective role of polyphenols interfering with NF-κB signaling in amyloidosis development. Polyphenols may also modulate and downregulate the proinflammatory properties of astrocytes, contributing to a reduction in inflammation in the brain. Rottlerin, a polyphenol from *Mallotus philippinensis*, being a known PKC-δ inhibitor, was reported to reduce matrix metalloproteinase 9 (MMP-9) release from astrocytes exposed to phorbol esters, and to inhibit astrocyte migration [145]. As MMP-9 activity is an important part of the increase in BBB permeability and inflammatory cell recruitment, rottlerin may be useful in the control of neuroinflammation. Plant polyphenols may also reduce the chemokine release from astrocytes. In a study on human primary astrocytes, Grabarczyk et al. reported that pre-exposure to quercetin or myricetin significantly reduced the chemokine release from astrocytes exposed to TNF-α, IL-1α, and C1q [146]. Plant polyphenols may also modulate the CREB signaling pathway in astrocytes and stimulate BDNF and GDNF synthesis, contributing to neurons’ survival and improvement of neural plasticity and memory [120,147,148,149,150,151].

### 3.3. Neuroprotective Mechanisms of Selected Polyphenols in Microglial Cells

Microglia are specialized immune cells that reside within the CNS. They play a crucial role in monitoring the CNS environment, providing protection against pathogens, and contributing to the maintenance of synapses and myelin [152]. Depending on extracellular signals, microglia can adopt distinct phenotypes, which reflect their functional state and impact CNS health [153]. Under physiological, homeostatic conditions, microglia exhibit a resting phenotype, often referred to as M0. In response to pro-inflammatory stimuli, such as LPS or interferon-gamma (IFN-γ), microglia become activated and shift toward a pro-inflammatory M1 phenotype. M1 microglia release a variety of inflammatory mediators, including TNF-α, IFN-γ, IL-1β, and IL-6, along with elevated production of NO and ROS. Although this inflammatory state is beneficial in the context of infection, prolonged activation can be neurotoxic, contributing to neuronal degeneration and the progression of neurodegenerative diseases [154,155]. Conversely, microglia can adopt an anti-inflammatory M2 phenotype in response to interleukin-4 (IL-4) and interleukin-13 (IL-13). M2 microglia exert their anti-inflammatory and reparative functions by secreting IL-10 and transforming growth factor-beta (TGF-β). They also support the maintenance of the extracellular matrix (ECM), promote wound healing, and exhibit enhanced phagocytic capacity. These characteristics endow M2 microglia with neuroprotective properties. Given the dualistic nature of microglial phenotypes, the modulation of microglial polarization from the M1 to the M2 state has emerged as a promising therapeutic strategy in the context of neuroinflammation and neurodegeneration [154,155].

#### 3.3.1. Influence of Curcumin on Microglia During the Course of Neurodegenerative Diseases

The positive effects of curcumin were shown in the study on APP/PS1 mice, which are AD animal models. Neuroinflammation contributing to the development of AD was lowered after treatment with curcumin, which targets PPAR-γ. The intensified activity of PPAR-γ caused by curcumin led to increased Aβ uptake by microglia and also lowered the activity of NF-κB, resulting in improved cognitive skills and inhibited neuroinflammation [156]. Microglia with an elevated amount of NF-κB are present in active demyelinating plaques occurring during the course of MS. The ability of curcumin to decrease the activity of NF-κB leads to the shift of microglial actions toward anti-inflammatory properties, which is visible in the lowered secretion of TNF-α, IL-1β, IL-6, and interleukin-8 (IL-8) [157]. A study conducted on mice with rotenone-induced PD also confirmed the usefulness of curcumin in the treatment. The activation of NLRP3 inflammasome in microglia leads to the secretion of interleukin-18 (IL-18) and IL-1β, resulting in PD onset. Curcumin turned out to exert inhibiting properties of NLRP3, protecting against PD development [158].

#### 3.3.2. Influence of Luteolin on Microglia During the Course of Neurodegenerative Diseases

Luteolin was proven to have anti-inflammatory properties, which are visible in suppressing microglia activation. On the molecular level, luteolin suppresses the activity of NF-κB, MAPK, and JAK-STAT, which are responsible for microglia activation and therefore neuroinflammation [159]. These properties were also confirmed in the study on dopaminergic neuron degeneration. Luteolin was tested in the conditions of the neuroinflammation induced by LPS in the culture of neurons with microglia. The addition of this polyphenol led to the reduced activity of microglia and inhibited the secretion of pro-inflammatory TNF-α, NO, and superoxide [160]. Toll-like receptor 4 (TLR4) present on microglia acts on NF-κB signaling, allowing these cells to take part in neuroinflammation. The results of a study on PD mouse models revealed that luteolin has the ability to suppress TLR4 activity, leading to a change in microglia phenotype from M1 to M2 [161]. Luteolin also turns out to have beneficial effects in the prevention and treatment of AD. Due to the complexity of AD pathogenesis, the focus on neuroinflammation is an increasingly popular approach [162]. Multiple studies confirmed the efficacy of luteolin in the context of inflammation in AD. As an example, a study on a mouse AD animal model showed that the intake of luteolin led to a decrease in TNF-α, IL-1β, NF-κB, and p65 levels [163].

#### 3.3.3. Influence of Resveratrol on Microglia During the Course of Neurodegenerative Diseases

Converting microglia to the M1 phenotype by LPS can lead to the activation of TLR4, which in turn switches on transcription factors such as NF-κB. As a result, pro-inflammatory cytokines are released. Resveratrol, similar to luteolin, acts against the effects of TLR4, leading to the inhibition of the NF-κB pathway. Also, the activity of the COX-2 pathway and NLRP3, both associated with inflammation, is reduced by resveratrol. This activity contributes to the increased presence of M2 microglia and, therefore, may be beneficial in MS treatment [164]. By mitigating the pro-inflammatory activity of microglia, resveratrol contributes to lowering the levels of TNF-α, IL-β, prostaglandin E2, cyclooxygenases, and iNOS. It is suggested that resveratrol influences microglia by acting on SIRT1, which leads to an intensified Th2 response, alleviating inflammation [165]. In a study on a rotenone-induced in vitro model of PD, resveratrol also acted as an anti-inflammatory by suppressing microglia activity. It was proven that resveratrol’s mechanism of action is focused on inhibiting the STAT1 pathway, which leads to the suppression of M1 microglia polarization [166]. Resveratrol is also involved in shifting microglia into the M2 phenotype by inducing increased secretion of IGF-1, exhibiting these properties [167].

#### 3.3.4. Influence of Quercetin on Microglia During the Course of Neurodegenerative Diseases

Another polyphenol with potential in the treatment of neuroinflammatory and neurodegenerative diseases by influencing microglia activity is quercetin. It was confirmed that this compound has the ability to shift the phenotype of microglia toward anti-inflammatory M2 [168]. The ability of quercetin to modify the polarization of microglia toward M2 was tested in a study with the use of mice models of AD. It was suggested that quercetin has the ability to activate the Nrf2/HO-1 pathway, which results in acquiring the M2 phenotype by microglia [169]. Additionally, after contact with quercetin, microglia were enabled to take in myelin debris. In a study on the lysophosphatidylcholine (LPC)-induced focal demyelination rat model, it was proven that the usage of quercetin led to the lowering of ionized calcium-binding adapter molecule 1 (lba1) levels, which is a microglia marker, indicating a decrease in their amount and therefore lower chances of the presence of inflammation [168]. The activation of the NLRP3 inflammasome is connected to the intensified secretion of inflammatory agents by microglia. In a study on LPS-induced BV2 cells, it was proven that the addition of quercetin resulted in the suppression of the excessive expression of proteins linked to NLRP3 inflammasome, such as NLRP3, caspase-1, and IL-1β [170].

### 3.4. Neuroprotective Mechanisms of Selected Polyphenols in OPCs and OLs

Oligodendrocytes (OLs) are highly specialized cells with high metabolic demand. They produce myelin internodes, responsible for fast impulse conduction in neurons. The myelin sheath is not just a cover present around the axons, but is also metabolically active [171]. Moreover, the communication of oligodendrocyte progenitor cells (OPCs) with other cells, such as neurons, astrocytes, and the BBB endothelium, is also an important function. This crosstalk involves signaling through trophic factors such as TGF-β, BDNF, and GDNF [172]. Alterations in oligodendroglia may thus impact the homeostasis of the CNS [172,173,174]. OLs are the most vulnerable cells in the CNS [175]. These cells are very susceptible to oxidative stress due to their high metabolic activity, high iron content, and low antioxidant potential and anti-apoptotic protein level [176,177,178]. The alterations in redox balance are one of the most potent factors responsible for OL damage. The main source of ROS is mitochondria, which regulate many physiological processes, like energy production, ion homeostasis, and anti-apoptotic mechanisms of OPCs [179]. Oligodendrocyte loss and myelin destruction leave axons vulnerable to degeneration and result in neurological deficits observed in many CNS diseases, like MS, Alzheimer’s disease, and stroke.

The support of remyelination and proper OPC and OL functions constitutes one of the major targets for therapeutic interventions in demyelinating and neurodegenerative diseases. Nowadays, several studies have indicated the great potential of various polyphenols in these processes, pointing to their anti-inflammatory, antioxidant, and neuroprotective mode of action. Below, we summarize the current knowledge of the impact of selected polyphenols on OPCs and OLs.

#### 3.4.1. Influence of Quercetin on OPCs and OLs During the Course of Neurodegenerative Diseases

The main function of flavonoids is their ability to scavenge ROS, the activation of antioxidant enzymes, and the inhibition of enzymes related to the production of free radicals and factors related to oxidative stress, like iNOS and NO [180,181]. Among various flavonoids, quercetin is known for having neuroprotective properties and biological effects on nervous tissue [182]. The neuroprotective feature of quercetin is mainly related to the anti-inflammatory and antioxidant potential of this compound [182]. Fan et al. showed the anti-inflammatory effect of quercetin on the necroptosis of oligodendrocytes after spinal cord injury (SCI) [183]. Necroptosis is often exacerbated after SCI, leading to severe neurological damage. It was shown that the administration of quercetin significantly reduced myelin and axonal loss, improving the functional recovery of the animals. The authors indicated the reduced loss of the myelin basic protein (MBP) and neurofilament 200 (NF200) in the white matter of SCI mice after treatment with this flavonoid. MBP is one of the main components of myelin sheaths, and NF200 is a structural component of the cytoskeleton, involved in axon development. Moreover, this study indicated the reduced expression of pro-inflammatory factors such as TNF-α and iNOS [183]. In another study, quercetin protected OPCs, reducing their apoptosis and increasing their proliferation and differentiation [184,185]. This effect was mediated via the PI3K/Akt signaling pathway [184]. Additionally, in a study conducted on rat oligodendrocytes (OLN-93 cells), it was observed that quercetin increased the survival of these cells by modulating NF-κB signaling and inducing the paraoxonase 2 pathway [186]. Moreover, it was shown that quercetin protected OPCs from oxygen/glucose deprivation injury in vitro via the PI3K/Akt pathway [184]. In a cuprizone-induced demyelination model, treatment with quercetin decreased demyelination in the corpus callosum [187]. It was also shown that quercetin-treated mice had higher MBP and Olig2 (the transcription factor specific to oligodendrocyte progenitors) protein expression, which was associated with increased remyelination [187]. Quercetin induced more Olig2+ cells, indicating that it promotes the proliferation of OPCs [188]. Moreover, it was shown that quercetin significantly upregulated MBP levels and downregulated the Inhibitor of DNA binding 2 (ID2), the inhibitor of MBP expression [188,189].

#### 3.4.2. Influence of Resveratrol and Curcumin on OPCs and OLs During the Course of Neurodegenerative Diseases

Experiments conducted on cuprizone-induced demyelination animal models indicated the protective effect of resveratrol. The main mechanisms of action of resveratrol are a reduction in oxidative stress, the improvement of mitochondrial function, and the regulation of NF-κB signaling. This compound also increases the differentiation of OPCs and the remyelination process inside cuprizone-induced white matter lesions [190]. In LPS-mediated cytotoxicity, resveratrol exerts a protective role on oligodendrocytes. This role is related to Nrf2 and HO-1 pathways. The HO-1 pathway is suggested to provide resistance against stressful conditions, such as oxidative stress and inflammation [191,192,193]. LPS via TLR4 affected the release of trophic factors by OPCs, like TGF-β, BDNF, and GDNF, and resveratrol has the potential to reverse these changes. Moreover, in this model, resveratrol decreased the level of ROS and re-established the level of GSH and glutamate cysteine ligase (GCL) (an enzyme required for GSH biosynthesis), acting through the Nrf2/HO-1 pathway. It also inhibited the NF-κB and hypoxia-inducible factor 1 alpha (HIF-1α) expression after LPS, protecting OPCs in a mechanism dependent on HO-1 [194,195].

Curcumin was shown to reduce demyelination in cuprizone-treated mice and re-establish the redox balance. In vitro, curcumin reduced the apoptosis of oligodendrocytes and lowered the level of pro-inflammatory factors, simultaneously increasing the level of anti-inflammatory molecules [196]. It was shown that curcumin in dendrosomal nanoparticles enhances oligodendrogenesis from neural stem cells (NSCs) and OPCs. This effect was dose-dependent. Moreover, it also induces remyelination in vivo via oligodendrogenesis promotion. This study also showed that such a formulation of curcumin elevated the remyelination capacity of transplanted NSCs through the promotion of their survival and oligodendrogenesis capacity [197]. It did not, however, have any impact on OPCs [197]. Moreover, in another study, curcumin showed the potential to improve mitochondrial activity in OPCs in vitro and to elevate cell differentiation via PPARγ activation, ERK1/2 phosphorylation, and increased PGC-1α expression [198].

#### 3.4.3. Influence of Phloretin and Ellagic Acid on OPCs and OLs During the Course of Neurodegenerative Diseases

Phloretin, a flavonoid present in apples and strawberries, markedly stimulated remyelination in ex vivo and in vivo animal models [199]. It was shown that this compound directly impacted OPCs’ maturation. Previous authors have indicated that the mechanism of phloretin action relies on being a direct ligand for the fatty acid-sensing nuclear receptor PPARγ. An elevated level of MBP and myelin proteolipid protein (PLP) mRNA was also observed, as well as an increased ratio of MBP to O4 (preoligodendrocyte marker) [199]. The positive impact of PPARγ on remyelination is associated with changes in cellular lipid metabolism, the regulation of oxidative stress, and an improvement of mitochondrial function [200].

Ellagic acid is a polyphenolic compound found in various vegetables and fruits, like pomegranates, strawberries, green tea, and blackberries [201]. Ellagic acid promotes OL maturation, decreases oligodendrocyte apoptosis, and reduces demyelination and axonal loss in experimental autoimmune encephalomyelitis (EAE), an experimental model of multiple sclerosis [202]. Through a reduction in the level of IFN-γ, ellagic acid reduces the expression of the death receptor (FAS) localized on the surface of oligodendrocytes, thus preventing their apoptosis [203,204]. Moreover, it was shown that this compound reduces oxidative stress by decreasing the level of IL-17 in oligodendrocytes. Through this action, ellagic acid facilitates the regeneration of myelin, improves the maturation of oligodendrocytes, and increases their survival [205]. Moreover, it can induce interleukin-11 (IL-11) expression, which reduces microgliosis and oligodendrocyte cell death, thus controlling OL loss [206].

#### 3.4.4. The Role of Other Polyphenols in OPCs and OLs Function During Neurodegenerative Diseases

There are several other polyphenols worth mentioning in the context of their impact on oligodendroglia. However, there is limited knowledge about their mode of action. Scutellarin, a flavonoid from herbal medication (*Erigeron breviscapus* Hand-Mazz), inhibits neural stem cells’ apoptosis and promotes the differentiation of NSCs into oligodendrocytes [207]. This compound also reversed the cuprizone-induced inhibition of OPC maturation into oligodendrocytes [207]. It is proposed that scutellarin acts via the inhibition of the MAPK signaling pathway [207]. Siddiqui et al. studied the anti-inflammatory potential of gallic acid and vanillic acid in an in vitro lysolecithin-induced model of demyelination [208]. Both compounds efficiently reduced the expression of COX-2 and NF-κB, as well as the glial fibrillary acidic protein (GFAP), a marker of reactive astrocytes [208]. Moreover, it was shown that both compounds not only reduced demyelination but also promoted myelin formation from immature oligodendrocytes, indicating that the neuroprotective effect of gallic acid and vanillic acid is linked to their anti-neuroinflammatory potential [208]. Studies conducted on the M03-13 cell line, an immortalized human oligodendrocyte-like cell, indicated that chlorogenic acid has antioxidant, anti-inflammatory, and anti-apoptotic potential. It was shown that this compound reduces intracellular superoxide ions, mitochondrial ROS, and NADPH oxidase (NOXs)/dual oxidase 2 (DUOX2) levels. Moreover, it was shown that chlorogenic acid induces the blockade of proliferation of these cells via the arrest of the cell cycle at the G0/G1 phase, driving them to differentiation, which results in elevated levels of MBP and PLP expression [209]. Arbutin improved myelin repair in the demyelinated optic chiasm via the attenuation of inflammation, astrocyte activation, and oxidative stress. This compound decreased the level of inflammatory cytokines and iNOS expression levels. Moreover, it increases anti-inflammatory cytokine levels and antioxidant mediators, like Nrf2 and HO-1. Also, elevated expression levels of MBP and Olig2 were observed after arbutin treatment [210].

## 4. Nanodelivery of Polyphenols: Liposomes as a Promising Platform

Nanocarrier platforms are an emerging and increasingly significant method of delivering therapeutic agents. These systems employ various types of molecules that serve as protective and facilitating carriers for the therapeutic cargo. Nanocarriers can be based on inorganic materials, such as mesoporous silica or metal nanoparticles [211]. On the other hand, a wide range of organic-based delivery systems exists, including polysaccharide-based carriers (e.g., using chitosan, sodium alginate), polymeric systems (using both natural and synthetic polymers), protein-based, and lipid-based platforms [212,213,214]. Depending on the physical and chemical properties of the materials used, nanocarriers can be engineered into structures with diverse forms and functionalities [215]. A notable example is that phospholipids can be used to construct various nanostructures, such as liposomes, micelles, and gels. The delivered compound (for instance, a polyphenol) can either be encapsulated within the nanocarrier structure or conjugated to it. Liposomes represent the encapsulation approach, whereas polymer–polyphenol complexes or polyplexes are examples of conjugated systems [215,216]. Among the various nanocarrier platforms, lipid-based nanocarriers stand out as a prominent approach for polyphenol delivery. Among the most widely studied are liposomes and their derivatives, including transfersomes, phytosomes, ethosomes, and niosomes [217,218]. Liposomes are nanoscale vesicles composed of one or more phospholipid bilayers, forming spherical structures typically ranging from 1 nm to 1000 nm in diameter. The biodegradable lipid bilayer encloses an aqueous core, which is biocompatible, non-toxic, and provides a stable environment for encapsulated polyphenols, protecting them from external degradation, digestive enzymes, and systemic toxicity, thereby enhancing their bioavailability [219]. The low stability and poor bioavailability of polyphenols pose significant challenges to their effective therapeutic application. Liposomes, however, offer a flexible platform that allows a wide range of modifications to optimize delivery. These modifications include:

- Membrane composition adjustments, such as the incorporation of cholesterol, which stabilizes and stiffens the lipid bilayer [220].
- Surface functionalization, e.g., polyethylene glycol (PEG) coating, to prolong circulation time and reduce immune clearance (stealth effect) [214].
- Targeting strategies, involving the conjugation of antibodies or ligands to the liposomal surface, which can direct the nanocarrier to specific tissues or cell types [221].

In the context of neurodegenerative diseases, such modifications are particularly valuable for enabling liposomes to cross the BBB and accumulate selectively in affected regions of the CNS [222]. Common ligands that enhance BBB penetration, such as mannose, transferrin, and cell-penetrating peptides, are considered both effective and safe [223,224,225,226]. Table 2 presents the key physicochemical parameters of conventional, PEGylated, and targeted liposomes. Interestingly, liposome functionalization does not lead to a significant increase in vesicle diameter and, therefore, does not impair their ability to penetrate cells.

Liposomes and other nanocarriers represent valuable tools for the delivery of polyphenols and therapeutic agents to specific organs, as they protect bioactive compounds from premature degradation and enhance their bioavailability at the target site. In general, liposomes are regarded as biocompatible and relatively safe; however, their physicochemical properties can significantly influence their interaction with biological systems. Notably, liposomes and other nanoparticles may interact with plasma proteins, activate the complement system, and exert cytotoxic effects, depending on their chemical composition [234,235,236]. Early in vitro studies have demonstrated that liposomal toxicity is largely composition-dependent [237,238]. Beyond direct toxicity, nanoparticles may also elicit non-specific immune responses, including macrophage and complement activation, as well as the induction of adaptive immunity against proteins adsorbed onto their surface, forming a so-called protein corona [236,239]. It has been postulated that nanoparticles may act as haptens, unable to trigger an immune response on their own but capable of doing so when conjugated with carrier proteins [239,240]. This poses an additional risk for the development of immune reactions directed against corona-associated proteins [239,241]. The immunogenic and toxic potential of liposomal carriers has been comprehensively addressed in recent reviews by Pondman et al. and Inglut et al. [239,242]. These abilities underscore the importance of careful consideration of liposome design, including the composition, size, dosage, and route of administration. Furthermore, there is a growing need to identify safer and more biocompatible nanocarriers and implement robust protocols for toxicity testing and the assessment of immune system activation in liposome and nanocarrier studies. These aspects are critical for the advancement of nanoparticle-based delivery systems and their clinical translation.

### Advances in Liposomal Nanocarriers for Brain-Targeted Polyphenol Therapy

Liposomes appear to be a promising nanocarrier for delivering polyphenols to the CNS. Among the selected carriers, they are the most extensively studied in the literature. Despite their limitations, they allow significant flexibility for optimization and functional modification. Cheng et al. demonstrated that EGCG-loaded liposomes significantly attenuate microglial activation in response to LPS [227]. In addition, these liposomes were tested in a mouse model of PD. EGCG-loaded liposomes reduced LPS-induced proinflammatory cytokine production and restored motor function. Notably, both in vitro and in vivo studies showed that the liposomal formulation of EGCG exhibited higher bioactivity compared to free EGCG [227]. Another polyphenol tested in liposomal formulations is curcumin, a natural compound with well-documented anti-inflammatory and anticancer properties. Additionally, curcumin has been shown to inhibit the aggregation of Aβ peptides, which are implicated in the pathogenesis of AD [228]. In a study by Taylor et al., [228] the potential of curcumin-loaded liposomes to inhibit Aβ fibrillar and/or oligomeric aggregation in vitro was investigated. Interestingly, the authors employed three distinct methods for the preparation of curcumin-loaded liposomes: a classical approach, in which curcumin was incorporated into the lipid phase during liposome formation, and two innovative strategies involving either the conjugation of curcumin with a lipid prior to formulation or the post-synthetic attachment of a curcumin derivative to preformed liposomes using a click reaction. The latter approach involved the use of a classical copper (I)-catalyzed azide–alkyne cycloaddition, resulting in vesicles referred to as click-curcumin liposomes. All three curcumin formulations demonstrated inhibitory effects on Aβ aggregation, with the “click-curcumin liposomes” exhibiting the most potent anti-aggregative activity. The surface functionalization of liposomes or liposome-derived nanocarriers represents a promising but underexplored strategy for polyphenol delivery to the CNS. This approach deserves further investigation, particularly in relation to bioavailability enhancement and BBB permeability [228]. At last, there are functional studies showing the effect of polyphenols and their formulations on an in vivo model. Additionally, they use the oral route of administration. For example, 14-day oral administration of either free resveratrol or resveratrol-loaded liposomes in a PD rat model significantly reduced certain tested parameters, such as total ROS, nigra cells loss/apoptosis, and abnormal rotational behavior. Notably, resveratrol in liposomal form demonstrated greater neuroprotective and antioxidant effects than free resveratrol, likely due to its enhanced bioavailability and more efficient CNS delivery [229]. The work of Priprem et al., concerning the anxiolytic and cognitive effects of quercetin in a rat model, surprisingly did not demonstrate superior efficacy for quercetin-loaded liposomes [243]. Indeed, their findings suggest that free quercetin exhibited significantly greater activity than its liposomal formulation. However, a critical factor potentially influencing this outcome lies in the disparate dosages employed. The dosage of free quercetin was established at 300 ng/kg, whereas the quercetin-loaded liposomes were administered at 20 μg/day. Notably, the authors themselves acknowledge, albeit in a different context, that the estimated dose of liposomes was approximately 6000-fold lower than that of the free quercetin [243]. Consequently, it would be imprudent to conclude that the lack of effect observed with quercetin-loaded liposomes is inherently due to the encapsulation within liposomes; rather, these data are likely not directly comparable owing to the substantial difference in administered doses. On the other hand, Ji-Min Lv et al. [244] reported a significant increase in the bioavailability of proanthocyanidins encapsulated in liposomes. Proanthocyanidins extracted from kiwi leaves were loaded into lecithin-based liposomes, with an encapsulation efficiency of 73.84%, and a more than 200% increase in the bioaccessibility of the tested polyphenols was observed [244].

Furthermore, beyond the oral administration route of liposomes, Priprem et al. also explored an intranasal delivery method [243]. Intriguingly, the same liposomal formulation that showed limited efficacy via the oral route demonstrated significantly enhanced activity when administered intranasally compared to free quercetin at the aforementioned 6000-fold lower dose (20 μg/day). While direct comparisons between oral and intranasal delivery dosages are inherently challenging, the presented results nonetheless highlight a potentially interesting and promising alternative strategy for quercetin delivery [243]. The intranasal route appears to be a promising approach for the delivery of polyphenols. Its primary advantages include bypassing the BBB, minimal invasiveness, the rapid onset of action, and reduced systemic side effects due to targeted delivery to the CNS [245]. Intranasal administration of polyphenols using nanocarriers is an innovative strategy, and the information currently available in the literature about this strategy is limited and not yet comprehensive. Based on the aforementioned example of EGCG and a study in which curcumin delivered intranasally in exosomes protected a murine model from LPS-induced neuroinflammation, it can be concluded that the intranasal route is a promising and worthwhile direction for further research [227,246]. Although some studies indicate toxicity associated with cationic liposomes (whereas neutral liposomes were well-tolerated) upon repeated intravenous administration, to the best of our knowledge, no studies have evaluated the safety or toxicity of intranasally administered liposomes containing polyphenols [247]. However, there is a study involving cargos other than polyphenols that reports very mild nasal toxicity following the intranasal administration of liposomes [226]. When considering the intranasal route for liposome delivery, encapsulated compounds may accumulate in the brain; thus, relevant central nervous system adverse effects should be assessed [248]. Many studies consider lipid-based nanocarriers as a single group and often draw conclusions about the safety of liposomes based on data from other approaches or formulations utilizing natural phospholipids [249,250]. Although the lipid carrier itself may exert some toxicity, it is essential to evaluate the toxicity of each liposomal formulation individually, as the cargo itself may vary in toxicity, and due to the lack of manufacturing standardization, liposomal nanocarriers composed of the same phospholipids and carrying the same cargo can still be produced using different methods and under different conditions, potentially leading to different biological effects.

To enhance the cargo accumulation in the CNS, nanocarriers can be coated with targeting ligands. The transferrin receptor is a popular target to enable nanocarrier internalization by brain endothelial cells and easier BBB crossing. In practice, liposomes can be functionalized with transferrin, a transferrin fragment containing the receptor-binding motif, or an anti-transferrin receptor antibody [251]. Transferrin-targeted liposomes loaded with various cargoes have been shown to be an effective platform for CNS delivery. Targeting the transferrin receptor significantly improved association and internalization compared to non-targeted liposomes [232,233,252,253]. Transferrin-targeted liposomes loaded with grape extract have been studied in the context of neuroinflammation associated with PD [232]. According to the authors, their liposomal formulation “demonstrated excellent BBB-crossing capacity.” Moreover, the efficacy of targeted liposomes was assessed in terms of inhibiting β3-tubulin depletion, enhancing cell viability, reducing α-synuclein aggregation, and lowering ROS levels. These parameters were examined using an in vitro rotenone-induced PD model, and all tested liposomes exerted a significant and beneficial effect on the evaluated markers [232]. In another study, resveratrol encapsulated in transferrin-targeted liposomes was examined as an anti-glioblastoma (GBM) agent. The authors demonstrated that this approach exhibited therapeutic properties by significantly increasing apoptosis and reducing tumor volume. Notably, resveratrol delivered via transferrin-targeted liposomes showed greater activity compared to its delivery via non-targeted liposomes [233]. The transferrin receptor is an appealing target for liposome-mediated delivery, but it is not specific to brain endothelial cells or GBM alone. To enhance specificity and precision in the delivery of polyphenols, multiple targets can be employed to design, for example, dual-targeted nanocarriers. Although polyphenols are commonly consumed through diet, their administration in higher doses may produce adverse effects. Therefore, restricting the uptake of nanocarriers to specific cells may be beneficial for improving their overall therapeutic profile. Dual-targeted liposomes have already been utilized for CNS delivery. An example is the study by Ying et al., which investigated the delivery of daunorubicin using liposomes conjugated with p-aminophenyl-α-D-manno-pyranoside and transferrin [254]. The dual-targeting strategy first aimed to facilitate BBB penetration and then to direct the drug specifically to GBM. The delivery approach was evaluated using in vitro C6 glioma cells and in vivo in C6 glioma-bearing rats. The researchers reported a 24.9% increase in the transport ratio across the BBB and a 54.7% reduction in glioma spheroid volume. Moreover, after crossing the BBB, the dual-targeted daunorubicin liposomes increased the inhibitory rate against C6 glioma cells to 64%. Tumor-bearing rats treated with the dual-targeted formulation exhibited a significantly higher median survival (22 days) compared to rats treated with free daunorubicin (17 days). These findings may facilitate the future development of dual-targeted liposomes for polyphenol delivery [254]. In another study, liposomes carrying EGCG were designed to target both the transferrin receptor and α-synuclein [253]. The targeting molecules used were anti-transferrin receptor antibodies to enhance BBB penetration and anti-α-synuclein antibodies to increase neuronal uptake. Key parameters such as stability, target affinity, EGCG release, and liposomal uptake by hCMEC/D3 cells were evaluated. The study demonstrated that the dual-targeted liposomes were functional and potentially suitable as targeted nanocarriers. Interestingly, the presence of the second antibody did not impair the uptake efficiency by endothelial cells. Although the authors focused on PD treatment perspectives, they did not assess the effects of EGCG delivered via their dual-targeted liposomes in PD in vitro or in vivo models [253].

## 5. Conclusions and Future Perspective

Globally, the average life expectancy is steadily increasing, and so are expectations regarding the quality of life, particularly among the elderly. CNS disorders, especially neurodegenerative and neuroinflammatory diseases, are closely associated with aging and represent a major challenge for modern medicine. Polyphenols have emerged as promising candidates to address these challenges. Numerous in vitro and in vivo studies cited in this review highlight the strong antioxidant properties of polyphenols and their ability to regulate key signaling pathways involved in oxidative stress responses, apoptosis, and inflammation. These compounds modulate immune responses by regulating the expression of pro-inflammatory cytokines (e.g., TNF-α, interleukins) and chemokines. Despite their broad activity across many cell types implicated in CNS pathologies, the full extent of polyphenols’ impact on diseased neural tissues remains incompletely understood. Neurodegenerative and neuroinflammatory disorders are multifactorial and complex, involving both resident and infiltrating cell populations, as well as interactions governed by the gut–brain axis. This complexity makes it difficult to develop standardized research models, which are needed in clinical trials. Another major obstacle is the poor bioavailability of polyphenols. Orally administered polyphenols are often unstable and poorly absorbed, with their bioactivity being significantly influenced by their solubility, sensitivity to environmental factors, and the harsh conditions of the gastrointestinal tract. Various nanodelivery platforms, such as the liposomes described in this review, have been proposed to overcome these limitations. Liposomes have attracted special attention due to their previous applications in medicine and their demonstrated safety profiles. Currently, liposomal formulations of polyphenols are actively investigated for delivery to the CNS. Liposomes offer a versatile delivery platform that can be tailored through structural and surface modifications to optimize therapeutic performance. However, this flexibility introduces challenges related to formulation optimization. Furthermore, establishing safety, regulatory compliance, and scalability for clinical translation remains a significant hurdle.

Several issues need to be addressed in the future concerning the development of polyphenol-based nanocarriers and their implementation in clinical studies—the development of simple and reproducible methods for nanocarrier generation with standardized protocols; in-depth studies regarding the quality and functionality of materials used in nanocarrier synthesis and their safety profile, stability, biodegradability, polyphenol-loading efficiency, traceability, and specific targeting ability; long-term toxicity; controlled release kinetics; efficient penetration of BBB; and renal clearance. It is also important to assess how these polyphenol-loaded nanocarriers interact with blood elements, the immunological system, and normal tissue, and how immune tolerance for polyphenol delivery strategies may be achieved. As polyphenols and polyphenol-loaded nanocarriers must be taken up for a period of time to fully elucidate their health-promoting potential, it is also important to study the effect and possible side effects of the buildup of nanoparticles in the human body. Moreover, the choice of administration route should also be carefully considered as it influences the biodistribution and efficacy of polyphenol-loaded nanoparticles. The impact of the pH of the environment should also be considered, as it can significantly alter the solubility and stability of polyphenol and the nanocarrier itself. To advance the usage of nanocarriers for polyphenol loading, several tools should be implemented to assess potential adverse effects, such as in silico toxicology models and in vitro and in vivo studies. Moreover, the selection of polyphenol itself, which is meant to be incorporated into the nanoparticle, should be carried out with caution and based on the health benefits potential of the selected compound and the mechanism of action. Also, the safety profile of the selected dosage and duration of treatment with the chosen polyphenol is an important factor.

In summary, although there is a growing body of preclinical evidence supporting the health-promoting effects of polyphenols in neurodegenerative and immune-related CNS disorders, many questions remain unresolved. While the molecular mechanisms of polyphenol activity in isolated cell types are increasingly well understood, there is still a pressing need to explore their effects within more complex biological systems. Nanocarriers offer a promising solution to overcome the bioavailability limitations of polyphenols, yet the field must strive to establish standardized protocols for in vitro and in vivo testing to facilitate reproducibility and translational success. Future research should focus on the optimal route and formulation of administration, the long-term safety of liposomal intake, and the development of effective strategies to facilitate blood–brain barrier penetration.

## Figures and Tables

**Table 1:** Selected polyphenols with known administration doses and their side effects.

Polyphenol (Dosage)	Adverse Effect	Study Type	Reference
Epigallocatechin gallate (379 mg/day for 3 months)	Decreased blood iron levels	Randomized controlled trials, obese patients	[58]
Quercetin (500 mg/day for 12 weeks)	Decreased blood iron levels	Randomized, double-blind, placebo-controlled study	[59]
Raspberry and strawberry extracts (50 μg/mL GAE ^1^)	Significant reduction in lipase activity	In vitro	[60]
Quercetin (minimal inhibitory concentration = 20–50 µg/mL); Naringenin and hesperetin (minimal inhibitory concentration ≥ 250 µg/mL)	Strong negative impact on physiological intestinal microbiota	In vitro	[61]
Green tea catechins (630 mg)	Significantly reduced systemic digoxin levels	In vivo human study	[62]
Grapefruit juice (8 oz, 3× a day for 6 days)	Increased felodipine availability due to CYP3A4 inhibition	In vivo human study	[63]
Isoflavones (approximately 600 µg/g for 5 weeks)	35-fold increase in plasma phytoestrogen levels; decreased body and prostate weight	In vivo rat model	[64]
Soy isoflavones (5 mg/kg)	Elevated blood pressure	Human case study	[65]
Genistein (270 mg/kg); quercetin (302 mg/kg)	Dose-dependent DNA double-strand break in hematopoietic cells	In vitro model	[66]

^1^ Gallic acid equivalent.

**Table 2:** Characteristics and effects of polyphenol-loaded liposomal formulations.

Liposome-Based Composition	Functionalization	Size (nm)	Load	Encapsulation Efficiency (%)	Effect	Model	Source
Phosphatidylserine	-	132.86 ± 2.05	EGCG	70.4	Increased anti-inflammatory and neuroprotective effects	In vitro/in vivo	[227]
Liposomes composed of L-α-phosphatidylcholine	-	155.2 ± 1.23	EGCG	55.4	Increased anti-inflammatory and neuroprotective effects	In vitro/in vivo	[227]
Liposomes composed of sphingomyelin	-	97.6 ± 3.1	Curcumin	1.3 mol% (with respect to total phospholipid content)	Inhibited formation of Aβ1–42 fibrils	In vitro	[228]
Liposomes composed of lecithin	-	146–585	Resveratrol	73.54%	Increased dopaminergic neuron protection	In vivo (rat PD model)	[229]
Liposomes composed of Lipoid S100	PEG ^1^	100.6 ± 0.7	Curcumin	87 ± 3	Increased bilayer stability; decreased polyphenol release	In vitro	[230]
Liposomes composed of phosphatidylcholine	PEG ^1^	107.4–116.1	*Copaifera sabulicola* leaves extract	81.89–86.30	Reduced glioma cell viability by 93%	In vitro	[231]
Liposomes composed of brain lipids	Anti-TfR Ab ^2^	133.0 ± 27.0	Polyphenol-rich grape pomace extracts	20.15 μg/mL of polyphenols in 1 mg/mL of vehicles	Increased protection against neurodegeneration	In vivo (rat PD model)	[232]
Liposomes composed of egg phosphatidylcholine	Transferrin moieties	211.2 ± 0.8	Resveratrol	70−75	Increased cytotoxicity and apoptosis in glioblastoma	In vitro/in vivo	[233]

^1^ Polyethylene Glycol (PEG); ^2^ Transferrin Receptor Antibody (TfR Ab).
